# Quantitative assessment of lncRNA *HOTAIR* polymorphisms and cancer risk in Chinese population: a meta-analysis based on 26,810 subjects

**DOI:** 10.18632/oncotarget.19776

**Published:** 2017-08-01

**Authors:** Xu Liu, Qiongyu Duan, Jian Zhang

**Affiliations:** ^1^ Department of Neurology, First Affiliated Hospital of China Medical University, Shenyang 110001, Liaoning, China; ^2^ Department of Oncology, Shengjing Hospital of China Medical University, Shenyang 110004, Liaoning, China; ^3^ Department of Cell Biology, Key Laboratory of Cell Biology, Ministry of Public Health, Key Laboratory of Medical Cell Biology, Ministry of Education, China Medical University, Shenyang 110122, China

**Keywords:** HOTAIR, polymorphism, cancer risk, Chinese population, meta-analysis

## Abstract

As a well-known long non-coding RNA, *HOTAIR* has been demonstrated to be involved in carcinogenesis and progression of various human cancers. Previous studies have investigated the potential association between *HOTAIR* polymorphisms and cancer risk in Chinese population. However, the results remain conflicting. Therefore, for the first time, we conducted a meta-analysis to derive a more precise estimation of these associations for Chinese. PubMed, Embase, CNKI and Wanfang databases were systematically searched. Odds ratios with 95% confidence intervals were applied to assess the association between rs920778, rs4759314, rs7958904, rs874945 and rs1899663 polymorphisms of *HOTAIR* and cancer susceptibility. Heterogeneity, sensitivity analysis and publication bias were conducted to measure the robustness of our findings. A total of 21 eligible studies comprising 12,278 cases and 14,532 controls were analyzed. The pooled data showed that rs920778 polymorphism was significantly associated with an increased cancer risk in all five genetic models in Chinese population. As for rs4759314 and rs874945 polymorphisms, similarly increased risks were found in specific genetic models and stratified groups. However, significant decreases in cancer risk were observed for rs7958904 in the total population, as well as in subgroup analyses. In addition, lack of association was detected between rs1899663 polymorphism and cancer susceptibility. In summary, our meta-analysis implicates possible relationship between *HOTAIR* polymorphisms and cancer risk in Chinese population.

## INTRODUCTION

Long non-coding RNAs (lncRNAs), as a type of transcribed RNAs which are longer than 200 nucleotides with no protein-coding capacity, were initially claimed to be a fake transcriptional noise [1, 2]. Nowadays, it is becoming clear that lncRNAs are involved in a wide range of biological regulation in the carcinogenesis and progression of various human cancers [3–6]. *HOTAIR*, an lncRNA located on chromosome 12q13.13, is coded from the homebox C gene (*HOXC*) locus [7]. It has been demonstrated that *HOTAIR* could specifically interact with polycomb repressive complex 2 (*PRC2*) and *LSD1*/*CoREST*/*REST* complex, in turn induce its relating methylation of histone H3K27 and demethylation of histone H3K4 respectively, and consequently result in the alteration of genes expression profile [8, 9]. The aberrant expression of *HOTAIR* has been reported in a variety of human cancers such as breast cancer, gastric cancer, colorectal cancer and liver cancer [10–13]. In addition, *HOTAIR* was also shown to be involved in the progression of multiple types of cancers, indicating that *HOTAIR* might serve as a useful biomarker for tumorigenesis and progression [8, 14–16].

Several single nucleotide polymorphisms (SNPs) located in *HOTAIR* locus have been identified [17, 18]. Among them, the rs920778, rs4759314, rs7958904, rs874945 and rs1899663 polymorphisms are common and widely studied. In 2014, Zhang et al. firstly reported the association between three *HOTAIR* polymorphisms and cancer risk in Chinese population [19]. From then on, increasing epidemiologic studies from Chinese population explored the association of the common polymorphisms in *HOTAIR* with the risk of cancers including gastrointestinal cancers [18–22], estrogen-dependent cancers (cervical cancer, ovarian cancer and breast cancer) [23–27], thyroid carcinoma [28] and osteosarcoma [29]. However, the results are inconsistent. Also, as individual studies with limited sample sizes are difficult to obtain reliable conclusions; further validation of the results is needed. Thus, to get a more precise conclusion, we conducted a meta-analysis involving all eligible studies published to date to estimate the association between *HOTAIR* polymorphisms and cancer risk in Chinese population. To our knowledge, this is the first meta-analysis which investigates the association for Chinese.

## RESULTS

### Study characteristics

**Figure 1:**
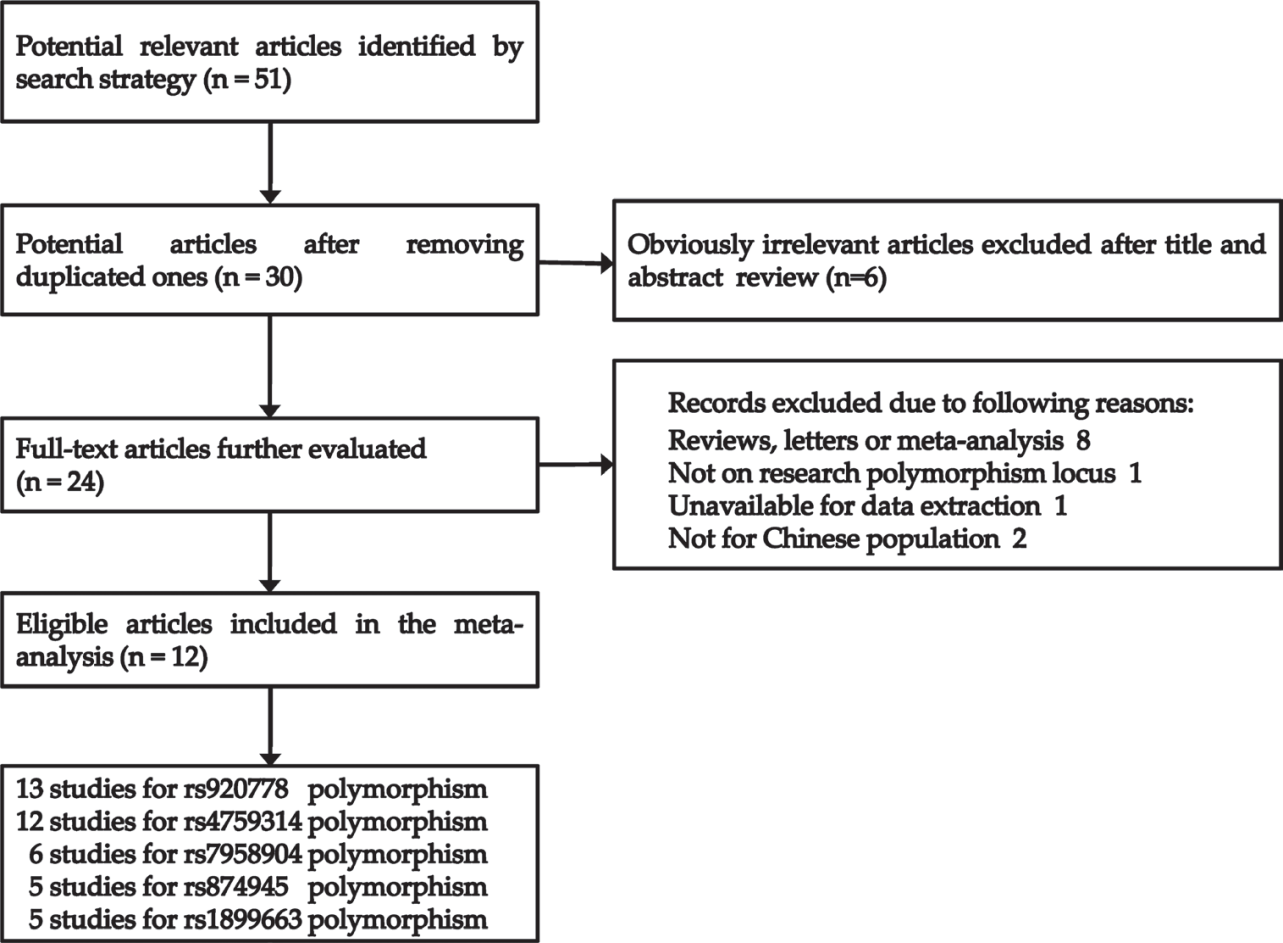
Flow diagram of the study selection process

The screening process of the studies was shown in Figure 1. A total of 51 relevant articles were initially retrieved by using our search strategy. After reviewing the titles and abstracts, 27 obviously irrelevant or duplicate articles were first excluded and 24 potential articles were left for further evaluation. Among these 24 articles, 8 reviews, letters or meta-analyses, 1 studies not on focus polymorphism locus [30], 1 study unavailable for data extraction [31] and 2 studies not relating to Chinese population [32, 33] were excluded. Finally, 12 eligible articles (21 studies) published from 2014 to 2016 were included in our meta-analysis. There are 13 studies available for rs920778 C>T polymorphism [19, 20, 25–28], 12 studies for rs4759314 A>G polymorphism [18–25, 28, 29], 6 studies for rs7958904 G>C polymorphism [18, 21, 24, 29], 5 studies for rs874945 G>A polymorphism [18, 21, 24, 29] and 5 studies for rs1899663 G>T polymorphism [19, 20, 23, 25, 28], respectively. The main characteristics and genotype distributions of all included studies were summarized in Supplementary Table 1.

### Quantitative analysis

### Meta-analysis for *HOTAIR* rs920778 C>T polymorphism

**Figure 2:**
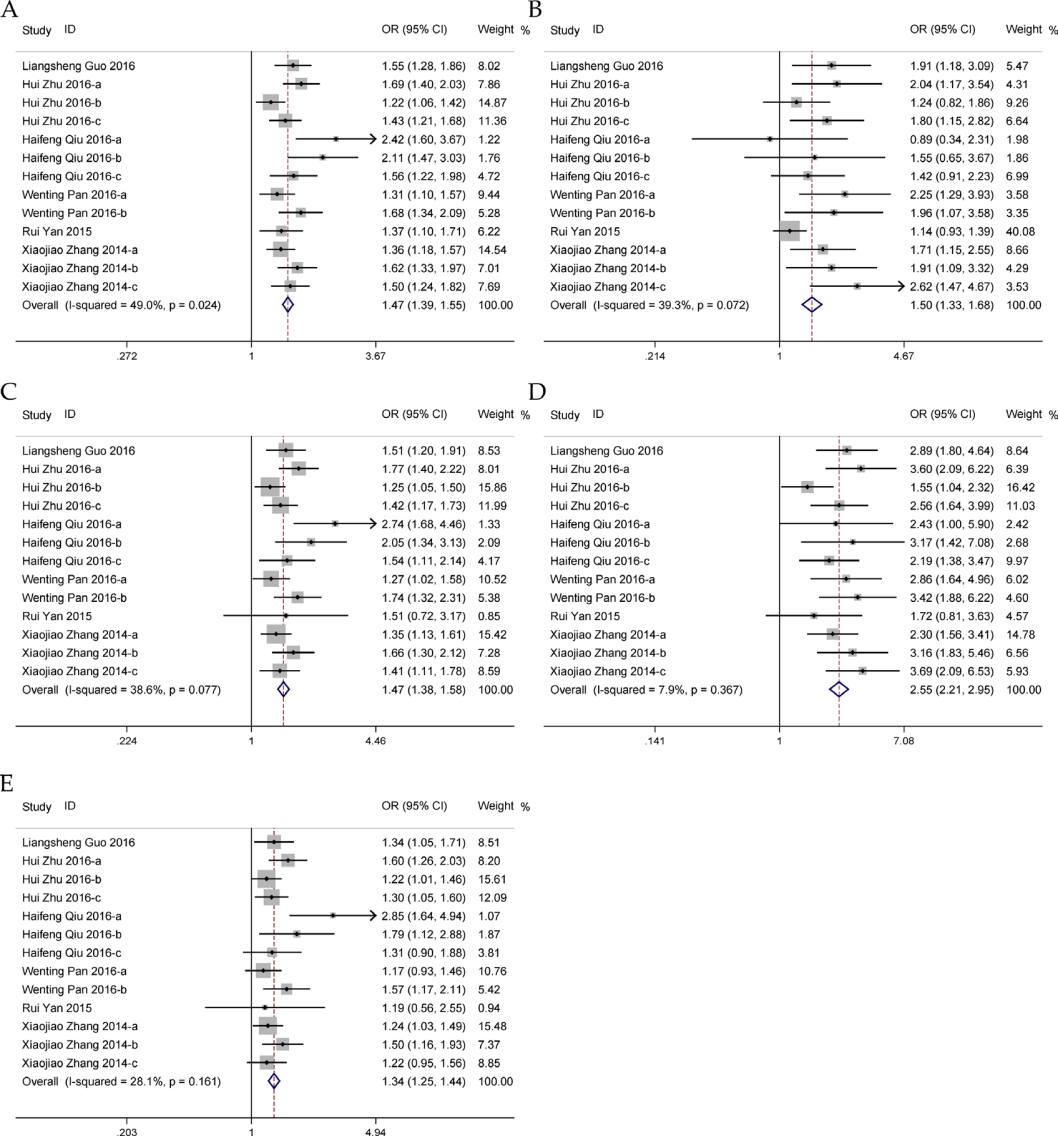
Forests for *HOTAIR* rs920778 polymorphism and cancer (**A**) allele model (T vs. C); (**B**) recessive model (TT vs. TC + CC); (**C**) dominant model (TC + TT vs. CC); (**D**) homozygous model (TT vs. CC); (**E**) heterozygous model (TC vs. CC).

Thirteen eligible studies including 6,854 cases and 8,477 controls were recruited in the meta-analysis. The results for the association between *HOTAIR* rs920778 polymorphism and cancer risk are presented in Table 1. The pooled analyses indicated that rs920778 polymorphism was significantly associated with an increased susceptibility of overall cancer in allelic, recessive, dominant, homozygous and heterozygous genetic models (Figure 2). The similar associations were observed both in estrogen-dependent cancers and gastrointestinal cancers when subsequently stratified by cancer type (Table 1). Analyses accounting for the source of controls in all five genetic models showed that rs920778 was remarkably associated with increased cancer risk in both population and hospital based groups in Chinese population (Table 1). And subgroup analyses based on the genotyping method also revealed similar results in RFLP and Taqman groups. In addition, we excluded four studies not satisfied with Hardy-Weinberg equilibrium (HWE) to reanalyze, and elevated risk was still found. Sensitivity analysis showed that the pooled ORs were not qualitatively changed by any single study in all five genetic models, which indicated that the results of our meta-analysis remained robust in Chinese population (Supplementary Figure 1). Visual inspection of funnel plot and Egger's test were performed to assess the publication bias. As shown in Supplementary Figure 2, the visual inspection of funnel plot revealed no obvious asymmetry but under allelic, dominant and heterozygous models. However, the results of Egger's test showed a publication bias only except homozygous model (T vs. C: *P* = 0.001; TT vs. TC+CC: *P* = 0.018; TC+TT vs. CC: *P* = 0.013; TT vs. CC: *P* = 0.215; TC vs. CC: *P* = 0.041).

**Table 1:** Summary ORs and 95% CIs of *HOTAIR* rs920778 polymorphism and cancer risk

Locus	N*	Allele (T vs. C)		Recessive (TT vs. TC+CC)		Dominant (TC+TT vs. CC)		Homozygote (TT vs. CC)		Heterozygote (TC vs. CC)	
		OR (95%CI) *P*	*I*^2^ (%)**	OR (95%CI) *P*	*I*^2^(%)**	OR (95%CI) *P*	*I*^2^ (%)**	OR (95%CI) *P*	*I*^2^ (%)**	OR (95%CI) *P*	*I*^2^ (%)**
Total	13	1.47 (1.39–1.55) *<* 0.01	49.0	1.50 (1.33–1.68) < 0.01	39.3	1.47 (1.38–1.58)< 0.01	38.6	2.55 (2.21–2.95) < 0.01	7.9	1.34 (1.25–1.44)< 0.01	28.1
Source of controls											
Population	5	1.42 (1.30–1.54) < 0.01	0	1.76 (1.23–2.51) 0.002	70.8	1.40 (1.25–1.56) < 0.01	0	2.70 (2.12–3.42) < 0.01	0	1.26 (1.12–1.41) < 0.01	0
Hospital	4	1.69 (1.50–1.91) < 0.01	44.8	1.71 (1.25–2.34) 0.001	0	1.71 (1.47–1.99) < 0.01	44.1	2.96 (2.18–4.02) < 0.01	0	1.64 (1.27–2.12) < 0.01	53.9
Method											
RFLP	9	1.43 (1.34–1.51) < 0.01	36.1	1.69 (1.36–2.10) < 0.01	54.5	1.43 (1.33–1.54) < 0.01	22.6	2.54 (2.15–3.01) < 0.01	33.6	1.31 (1.21–1.42) < 0.01	0
Taqman	3	1.92 (1.46–2.53) < 0.01	51.3	1.35 (0.93–1.94) 0.114	0	1.89 (1.50–2.38) < 0.01	47.7	2.40 (1.67–3.45) < 0.01	0	1.81 (1.17–2.80) 0.008	63.1
MOLDI-TOF-MS	1	1.55 (1.28–1.86) < 0.01	NA	1.91 (1.18–3.09) 0.009	NA	1.52 (1.20–1.91) < 0.01	NA	2.89 (1.80–4.64) < 0.01	NA	1.34 (1.05–1.71) 0.019	NA
Type of cancer											
Estrogen-dependent	5	1.66 (1.40–1.97) < 0.01	51.5	1.25 (1.06–1.48) 0.008	17.6	1.68 (1.43–1.97) < 0.01	30.8	2.44 (1.86–3.19) < 0.01	0	1.48 (1.24–1.75) < 0.01	46.5
Gastrointestinal	5	1.45 (1.34–1.58) < 0.01	18.3	2.00 (1.59–2.53) < 0.01	0	1.43 (1.30–1.58) < 0.01	19.0	2.89 (2.30–3.64) < 0.01	0	1.30 (1.17–1.44) < 0.01	5.6
Controls in HWE	9	1.45 (1.37–1.54) < 0.01	33.7	1.66 (1.35–2.05) < 0.01	51.4	1.46 (1.35–1.57) < 0.01	12.2	2.56 (2.17–3.02) < 0.01	34.1	1.33 (1.23–1.44) < 0.01	0

* Numbers of comparisons. RFLP: Restriction Fragment Length Polymorphism. MOLDI-TOF-MS: Matrix-Assisted Laser Desorption/ Ionization Time of Flight Mass Spectrometry. HWE: Hardy-Weinberg Equilibrium. NA: not available.

### Meta-analysis for *HOTAIR* rs4759314 A>G polymorphism

**Figure 3:**
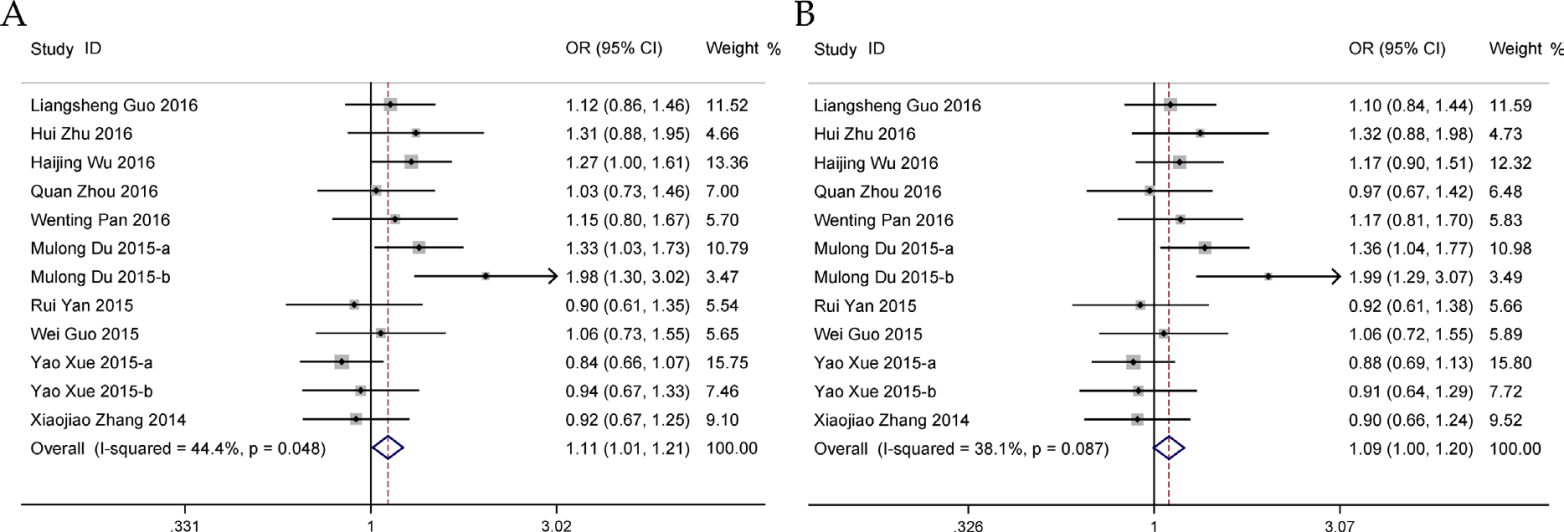
Forests for *HOTAIR* rs4759314 polymorphism and cancer (**A**) dominant model (GA + GG vs. AA); (**B**) heterozygous model (GA vs. AA).

Twelve studies comprising 8,136 cases and 9,472 controls reported the association of rs4759314 polymorphism with cancer susceptibility. As shown in Table 2, a significant association between *HOTAIR* rs4759314 polymorphism and cancer risk was observed in dominant and heterozygous models for Chinese (Figure 3). When different cancer types were considered, increased susceptibility was found only in estrogen-dependent cancer group (Table 2). Subsequent subgroup analyses also revealed significant association in the genotyping method of MALDI-TOF-MS group as well as age and sex matched group (Table 2). Publication bias was evaluated by the visual inspection of funnel plot and Egger's test, and there was no publication bias detected (G vs. A: *P* = 0.827; GG vs. GA+AA: *P* = 0.099; GA+GG vs. AA: *P* = 0.615; GG vs. AA: *P* = 0.069; GA vs. AA: *P* = 0.488; Supplementary Figure 3). However, further sensitivity analysis revealed that omission of each study made some significant differences on the findings (Supplementary Figure 4).

**Table 2:** Summary ORs and 95% CIs of *HOTAIR* rs4759314 polymorphism and cancer risk

Locus	N*	Allele (G vs. A)		Recessive (GG vs. GA+AA)		Dominant (GA+GG vs. AA)		Homozygote (GG vs. AA)		Heterozygote (GA vs. AA)	
		OR (95%CI) *P*	*I*^2^(%)	OR (95%CI) *P*	*I*^2^(%)	OR (95%CI) *P*	*I*^2^(%)	OR (95%CI) *P*	*I*^2^(%)	OR (95%CI) *P*	*I*^2^(%)
Total	12	1.11 (0.99–1.26) 0.087	50.4	1.11 (0.80–1.55) 0.532	0	1.11 (1.01–1.21) 0.026	44.4	1.31 (0.95–1.80) 0.096	0	1.09 (1.00–1.20) 0.058	38.1
Source of controls											
Population	4	1.00 (0.84–1.19) 0.962	0	0.93 (0.29–3.00) 0.898	0	1.00 (0.83–1.20) 0.977	0	0.93 (0.29–2.99) 0.901	0	1.00 (0.83–1.20) 0.990	0
Hospital	6	1.13 (0.92–1.38) 0.259	68.7	0.96 (0.60–1.53) 0.862	17.2	1.13 (0.91–1.41) 0.256	67.3	1.07 (0.68–1.67) 0.782	25.1	1.13 (0.91–1.40) 0.273	65.4
Method											
RFLP	5	1.04 (0.89–1.22) 0.629	0	0.89 (0.32–2.45) 0.817	0	1.04 (0.89–1.23) 0.604	0	0.95 (0.35–2.60) 0.925	0	1.05 (0.89–1.24) 0.589	0
Taqman	4	1.15 (0.83–1.61) 0.403	81.1	0.59 (0.28–1.28) 0.184	34.6	1.18 (0.83–1.66) 0.356	80.2	0.69 (0.33–1.45) 0.325	48.4	1.19 (0.85–1.66) 0.317	78.5
MALDI-TOF-MS	3	1.21 (1.05–1.39) 0.009	0	1.39 (0.92–2.09) 0.116	0	1.17 (1.00–1.36) 0.055	0	1.64 (1.12–2.41) 0.012	0	1.10 (0.93–1.30) 0.264	0
Type of cancer											
Estrogen-dependent	3	1.18 (1.02–1.37) 0.022	42.2	1.36 (0.86–2.14) 0.192	0	1.15 (0.98–1.35) 0.091	4.7	1.66 (1.08–2.55) 0.022	0	1.09 (0.92–1.30) 0.303	0
Gastrointestinal	5	1.09 (0.90–1.33) 0.369	64.3	0.69 (0.35–1.34) 0.270	0	1.11 (0.90–1.36) 0.326	63.5	0.77 (0.41–1.48) 0.437	5.3	1.11 (0.91–1.36) 0.295	61.7
Age and sex matched	11	1.12 (0.98–1.28) 0.099	54.8	1.11 (0.79–1.56) 0.540	0	1.11 (1.01–1.22) 0.027	49.4	1.31 (0.95–1.81) 0.098	0	1.10 (1.00–1.21) 0.060	43.7
Controls in HWE	10	1.09 (0.95–1.26) 0.237	51.8	0.84 (0.51–1.38) 0.493	0	1.10 (0.95–1.28) 0.200	50.4	0.94 (0.58–1.53) 0.815	0	1.09 (0.99–1.21) 0.092	47.6

* Numbers of comparisons. RFLP: Restriction Fragment Length Polymorphism. MOLDI-TOF-MS: Matrix-Assisted Laser Desorption/ Ionization Time of Flight Mass Spectrometry. HWE: Hardy-Weinberg Equilibrium.

### Meta-analysis for *HOTAIR* rs7958904 G>C polymorphism

**Figure 4:**
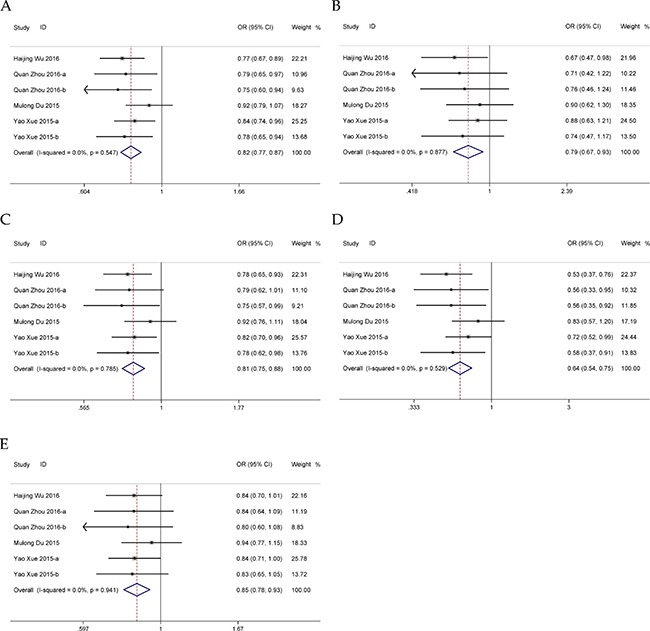
Forests for *HOTAIR* rs7958904 polymorphism and cancer (**A**) allele model (C vs. G); (**B**) recessive model (CC vs. CG + GG); (**C**) dominant model (CG + CC vs. GG); (**D**) homozygous model (CC vs. GG); (**E**) heterozygous model (CG vs. GG).

A total of six studies consisting of 4,387 cases and 4,812 controls were included in the quantitative analysis. No significant heterogeneity was detected across studies in all five genetic models and the fixed-effects model was therefore selected to summarize the ORs. Overall, a decreased risk of cancer was observed for Chinese (Table 3, Figure 4). When stratified by the type of cancers, a similarly decreased risk was identified in gastrointestinal cancer group (Table 3). Moreover, according to the results of subsequent subgroup analyses, the decreased risk does not obviously influenced by genotyping method and HWE status (Table 3). Sensitivity analysis revealed that the pooled ORs were not conspicuously changed by any single study (Supplementary Figure 5). No publication bias was detected in all genetic models (C vs. G: *P* = 0.362; CC vs. CG+GG: *P* = 0.305; CG+CC vs. GG: *P* = 0.416; CC vs. GG: *P* = 0.298; CG vs. GG: *P* = 0.523; Supplementary Figure 6).

**Table 3:** Summary ORs and 95% CIs of *HOTAIR* rs7958904 polymorphism and cancer risk

Locus	N*	Allele (C vs. G)		Recessive (CC vs. CG+GG)		Dominant (CG+CC vs. GG)		Homozygote (CC vs. GG)		Heterozygote (CG vs. GG)	
		OR (95%CI) *P*	*I*^2^(%)	OR (95%CI) *P*	*I*^2^(%)	OR (95%CI) *P*	*I*^2^(%)	OR (95%CI) *P*	*I*^2^(%)	OR (95%CI) *P*	*I*^2^(%)
Total	6	0.82 (0.77–0.87) < 0.01	0	0.79 (0.67–0.93) < 0.01	0	0.81 (0.75–0.88)< 0.01	0	0.64 (0. 54–0.75) < 0.01	0	0.85 (0.78–0.93) < 0.01	0
Method											
Taqman	3	0.85 (0.78–0.93) < 0.01	0	0.85 (0.69–1.06) 0.143	0	0.84 (0.76–0.94) < 0.01	0	0.72 (0.58–0.89) < 0.01	0	0.87 (0.78–0.98) 0.017	0
MOLDI-TOF-MS	3	0.77 (0.70–0.86) < 0.01	0	0.71 (0.54–0.91) < 0.01	0	0.78 (0.68–0.88) < 0.01	0	0.55 (0.42–0.70) < 0.01	0	0.83 (0.73–0.95) < 0.01	0
Type of cancer											
Estrogen-dependent	1	0.77 (0.67–0.89) < 0.01	NA	0.67 (0.47–0.98) 0.037	NA	0.78 (0.65–0.93)< 0.01	NA	0.53 (0.37–0.76) < 0.01	NA	0.84 (0.70–1.01) 0.063	NA
Gastrointestinal	3	0.85 (0.78–0.93) < 0.01	0	0.85 (0.69–1.06) 0.143	0	0.84 (0.76–0.94) < 0.01	0	0.72 (0.58–0.89) < 0.01	0	0.87 (0.78–0.98) 0.017	0
Controls in HWE	5	0.83 (0.77–0.89) < 0.01	0	0.79 (0.66–0.94) < 0.01	0	0.82 (0.75–0.90) < 0.01	0	0.65 (0.55–0.77) < 0.01	0	0.86 (0.78–0.94) < 0.01	0

* Numbers of comparisons. MOLDI-TOF-MS: Matrix-Assisted Laser Desorption/ Ionization Time of Flight Mass Spectrometry. HWE: Hardy-Weinberg Equilibrium. NA: not available.

### Meta-analysis for *HOTAIR* rs874945 G>A polymorphism

**Figure 5:**
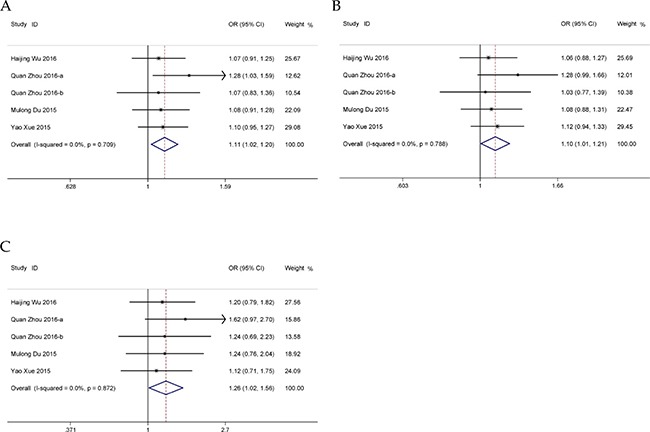
Forests for *HOTAIR* rs874945 polymorphism and cancer (**A**) allele model (A vs. G); (**B**) dominant model (AG + AA vs. GG); (**C**) homozygous model (AA vs. GG).

Five eligible case-control studies with 3,800 cases and 4,160 controls were included in the meta-analysis. Overall, we found a significant association between rs874945 G>A polymorphism and cancer risk in allelic, dominant and homozygous models (Table 4, Figure 5). Stratification accounting for genotyping method revealed increased cancer risk existed in MALDI-TOF-MS genotyping method group (Table 4). Further subgroup analyses of cancer type and HWE status were conducted and no significant result was observed (Table 4). Sensitivity analysis indicated that the pooled ORs remained robust only in recessive and heterozygous models (Supplementary Figure 7). No publication bias was detected in all genetic models (A vs. G: *P* = 0.495; AA vs. AG+GG: *P* = 0.362; AG+AA vs. GG: *P* = 0.715; AA vs. GG: *P* = 0.503; AG vs. GG: *P* = 0.962; Supplementary Figure 8).

**Table 4:** Summary ORs and 95% CIs of *HOTAIR* rs874945 polymorphism and cancer risk

Locus	N*	Allele (A vs. G)		Recessive (AA vs. AG+GG)		Dominant (AG+AA vs. GG)		Homozygote (AA vs. GG)		Heterozygote (AG vs. GG)	
		OR (95%CI) *P*		*I*^2^(%)		OR (95%CI) *P*		*I*^2^(%)		OR (95%CI) *P*		*I*^2^(%)		OR (95%CI) *P*		*I*^2^(%)		OR (95%CI) *P*		*I*^2^(%)	
Total	5	1.11 (1.02–1.20) 0.013	0	1.14 (0.91–1.42) 0.262	0	1.10 (1.01–1.21) 0.039	0	1.26 (1.02–1.56) 0.035	0	1.08 (0.98–1.19) 0.120	0
Method											
Taqman	2	1.09 (0.98–1.22) 0.132	0	1.07 (0.76–1.50) 0.717	0	1.10 (0.97–1.25) 0.148	0	1.17 (0.84–1.64) 0.353	0	1.09 (0.96–1.25) 0.199	0
MOLDI-TOF-MS	3	1.12 (1.00–1.26) 0.046	0	1.19 (0.89–1.60) 0.245	0	1.11 (0.97–1.27) 0.139	0	1.33 (1.00–1.76) 0.050	0	1.07 (0.93–1.23) 0.365	0
Type of cancer											
Estrogen-dependent	1	1.07 (0.91–1.25) 0.419	NA	1.14 (0.74–1.75) 0.551	NA	1.06 (0.88–1.27) 0.568	NA	1.20 (0.79–1.82) 0.383	NA	1.03 (0.85–1.26) 0.749	NA
Gastrointestinal	2	1.09 (0.98–1.22) 0.132	0	1.07 (0.76–1.50) 0.717	0	1.10 (0.97–1.25) 0.148	0	1.17 (0.84–1.64) 0.353	0	1.09 (0.96–1.25) 0.199	0
Controls in HWE	2	1.09 (0.98–1.22) 0.132	0	1.07 (0.76–1.50) 0.717	0	1.10 (0.97–1.25) 0.148	0	1.17 (0.84–1.64) 0.353	0	1.09 (0.96–1.25) 0.199	0

^*^Numbers of comparisons. MOLDI-TOF-MS: Matrix-Assisted Laser Desorption/ Ionization Time of Flight Mass Spectrometry. HWE: Hardy-Weinberg Equilibrium. NA: not available.

### Meta-analysis for *HOTAIR* rs1899663 G>T polymorphism

Five studies with 3,112 cases and 3,817 controls were included to estimate the association between rs1899663 G>T polymorphism and cancer risk. The results of this meta-analysis were shown in Table 5, and no significant association was identified in all five genetic models.

**Table 5:** Summary ORs and 95% CIs of *HOTAIR* rs1899663 polymorphism and cancer risk

Locus	N*	Allele (T vs. G)		Recessive (TT vs. TG+GG)		Dominant (TG+TT vs. GG)		Homozygote (TT vs. GG)		Heterozygote (TG vs. GG)	
		OR (95%CI) *P*	*I*^2^(%)	OR (95%CI) *P*	*I*^2^ (%)	OR (95%CI) *P*	*I*^2^ (%)	OR (95%CI) *P*	*I*^2^ (%)	OR (95%CI) *P*	*I*^2^ (%)
Total	5	0.93 (0.84–1.02) 0.102	0	0.78 (0.54–1.11) 0.163	0	0.93 (0.84–1.04) 0.193	0	0.73 (0.51–1.03) 0.071	0	0.95 (0.85–1.06) 0.350	0
Source of controls											
Population	3	0.93 (0.83–1.04) 0.208	0	0.79 (0.52–1.20) 0.265	0	0.94 (0.82–1.07) 0.334	0	0.74 (0.49–1.11) 0.147	0	0.96 (0.83–1.10) 0.512	0
Hospital	1	1.05 (0.84–1.31) 0.654	NA	0.82 (0.33–2.02) 0.658	NA	1.08 (0.84–1.39) 0.548	NA	0.88 (0.36–2.15) 0.778	NA	1.09 (0.85–1.41) 0.494	NA
Method											
RFLP	4	0.90 (0.81–1.00) 0.044	0	0.77 (0.52–1.13) 0.184	0	0.90 (0.80–1.02) 0.086	0	0.70 (0.48–1.02) 0.066	0	0.92 (0.82–1.04) 0.176	0
MOLDI-TOF-MS	1	1.05 (0.84–1.31) 0.654	NA	0.82 (0.33–2.02) 0.658	NA	1.08 (0.84–1.39) 0.548	NA	0.88 (0.36–2.15) 0.778	NA	1.09 (0.85–1.41) 0.494	NA
Type of cancer											
Estrogen-dependent	2	0.96 (0.82–1.13) 0.620	23.1	0.78 (0.45–1.37) 0.395	0	0.98 (0.82–1.17) 0.822	18.0	0.75 (0.43–1.29) 0.296	0	1.00 (0.83–1.21) 0.991	0
Gastrointestinal	2	0.95 (0.83–1.09) 0.443	0	0.80 (0.47–1.35) 0.400	0	0.96 (0.82–1.12) 0.576	0	0.77 (0.46–1.29) 0.325	0	0.97 (0.83–1.14) 0.729	0

* Numbers of comparisons. MOLDI-TOF-MS: Matrix-Assisted Laser Desorption/ Ionization Time of Flight Mass Spectrometry. RFLP: Restriction Fragment Length Polymorphism. NA: not available.

## DISCUSSION

Cancer is a polygenic and multifactorial disease which is thought to be caused by complex genetic factors and gene-environment interactions. The lncRNA polymorphisms have been demonstrated to be involved in carcinogenesis and progression of different types of cancers. Recently, the association between lncRNA *HOTAIR* polymorphisms and cancer risk has been developed gradually. However, the results remain contradictory and inconclusive, especially in different geographical location and ethnicity groups. In 2016, three similar meta-analyses involving eight articles explored the association of *HOTAIR* polymorphisms with several kinds of cancers among worldwide population [34–36]. However, none of them drew a conclusion for Chinese population. Since then, six new articles with nine individual studies for Chinese have been published [23, 24, 26–29]. Therefore, it is necessary to summarize all eligible individual studies and conduct a comprehensive meta-analysis to determine the correlations of *HOTAIR* polymorphisms with cancer susceptibility in Chinese population.

To the best of our knowledge, this is the first meta-analysis which attempt to explore the association between *HOTAIR* polymorphisms and cancer risk in population of Chinese ethnicity. A total of twenty-one eligible studies comprising 12,278 cases and 14,532 controls were included into current meta-analysis and it provided the most comprehensive assessment of the correlations of five common polymorphisms in the *HOTAIR* gene with cancer risk for Chinese to date.

Overall, our results demonstrated that rs920778 polymorphism of *HOTAIR* was significantly associated with increased cancer risk among Chinese population. Compared with those previous meta-analyses, some advantages of our analysis should be highlighted. First, although our combined results were consistent with most individual studies and meta-analyses, we definitely expended the sample size and boosted the statistical power dramatically by adding another seven individual studies with 7,119 subjects on the basis of the updated data. Second, we conducted a more comprehensive subgroup analysis. Noteworthy, in the analysis based on cancer type, we observed an increased risk for this polymorphism both in estrogen-dependent cancer group and gastrointestinal cancer group. Third, we conducted all the analyses for five genetic models including allelic, recessive, dominant, homozygous and heterozygous models to draw a comprehensive assessment. Fourth, when we excluded the studies involving Caucasian population, the heterogeneity of the analyses was significantly decreased. That helped to enhance the reliability of our conclusion.

As for rs4759314 polymorphism, even though no association was found in previous meta-analyses [34–36], the evidence from our meta-analysis supported a significant association with increased cancer risk in dominant and heterozygous models for Chinese. The discrepancy between our findings and previous meta-analyses might be due to the inclusion of another four recent studies involving 5,423 subjects in our analysis. Noteworthy, further subgroup analyses by cancer type revealed that significantly increased risks were only found in the estrogen-dependent cancer group, and it suggested the increased cancer risk might be tumor type-specific. Conversely, rs7958904 polymorphism exhibited as a potential protective factor for cancer risk on the basis of our results that this polymorphism was associated with the decreased cancer susceptibility in Chinese population. And the associations were further confirmed by stratified analyses according to genotyping method, cancer type and HWE status.

In addition, our pooled analyses also showed a significantly increased cancer risk for rs874945 polymorphism in allelic, dominant and homozygous models. However, potentially due to the relatively small sample size with only 5 studies involved, the elevated cancer risk for rs874945 did not exhibit tumor type specificity. So these results should be interpreted with caution and further studies are needed to clarify the accurate association. Lastly, as for rs1899663 polymorphism, our results could not provide any evidence of such an association with cancer risk under any genetic model.

There are a few potential limitations existed in the present meta-analysis. First, all published articles retrieved were written in English in our study, which may cause potential language bias. Second, the estimations about interactions between gene-gene, gene-environment, and multiple polymorphic loci in the same gene were not performed. Third, most of the studies included in our meta-analysis were concerning estrogen-dependent and gastrointestinal cancers. Thus this limited the general application of the results to other types of cancers, such as lung cancer, liver cancer and so on. Fourth, sensitivity analyses for rs4759314 and rs874945 polymorphisms revealed that omission of each study made some significant changes on the findings, which could be explained by the limited number of studies involved. Finally, as for rs920778, most of the studies included reported positive results and the publication bias was detected except in homozygous model. Thus, the comprehensive analyses should be interpreted with caution.

In summary, the current meta-analysis provides evidence that four functional polymorphisms of *HOTAIR* involving rs920778, rs7958904, rs4759314, and rs874945 might contribute to genetic susceptibility to cancer risk in Chinese population, whereas rs1899663 may have no impact. Accordingly, large scale and well-designed studies are warranted to confirm the association of above polymorphisms in *HOTAIR* and cancer risk in the future.

## MATERIALS AND METHODS

### Literature search strategy

Eligible studies regarding the association between *HOTAIR* polymorphisms and cancer risk in Chinese population were systematically searched from Pubmed, Embase, Wanfang and CNKI databases up to October 31, 2016. The terms used for search were as follows: “HOX transcript antisense RNA OR *HOTAIR*” and “polymorphism OR variant OR SNP OR genotype OR allele” and “cancer OR carcinoma OR tumor OR neoplasm”. In addition, citation lists of all relevant articles were manually searched for additional eligible publications.

### Inclusion and exclusion criteria

Studies were all selected according to the following criteria: (1) case-control design study; (2) evaluating the association between *HOTAIR* polymorphisms and cancer risk in Chinese population; (3) available genotype distribution data for calculating the odds ratios (ORs) with 95% confidence intervals (CIs). Reviews, letters, conference abstracts, duplicate studies and studies without sufficient genotype information were excluded. In addition, we finally selected the study with larger sample size from duplicate publications.

### Data extraction

The following data were extracted by two independent investigators: first author, publication year, source of controls, sample size, genotype frequency, genotyping methods, age and sex matched status, type of cancers, *P* value for Hardy-Weinberg equilibrium (HWE) in the control group. Disputes were resolved through group discussion.

### Statistical analysis

The crude ORs with 95% CIs were calculated to determine the relationship between the *HOTAIR* polymorphisms and cancer susceptibility in Chinese population. For the rs4759314 polymorphism, the pooled ORs were estimated by allelic (G vs. A), recessive (GG vs. GA+AA), dominant (GA+GG vs. AA), homozygous (GG vs. AA) and heterozygous (GA vs. AA) models. As for rs920778, rs7958904, rs874945, and rs1899663 polymorphisms, similar five genetic models were assessed. Subgroup analyses based on source of controls, type of cancers, genotyping methods, HWE status of controls and case-control matched status were subsequently performed. HWE was examined by chi-square test in the controls. The *I*^2^ statistic was used to evaluate the heterogeneity between studies. If *I*^2^ > 50%, significant heterogeneity was found and the random-effects model should be applied. Otherwise, the fixed-effects model should be used. Sensitivity analysis was conducted by sequentially omitting each single study to evaluate the stability of our results. Publication bias was assessed by both visual inspection of funnel plot and Egger's test. The STATA software 12.0 (StataCorp LP, College Station, TX) was used for all the statistical analyses. *P* < 0.05 was considered to be statistically significant.

## SUPPLEMENTARY MATERIALS FIGURES AND TABLES




