# Supplementary material for: Quantitative assessment of lncRNA HOTAIR polymorphisms and cancer risk in Chinese population: a meta-analysis based on 26,810 subjects
Source: Oncotarget. 2017 Aug 1;8(35):59698–708. doi: 10.18632/oncotarget.19776 (PMC5601769; doi:10.18632/oncotarget.19776)
Supplement: Supplementary file 1 [file oncotarget-08-59698-s001.pdf]

Quantitative assessment of lncRNA *HOTAIR* polymorphisms and cancer risk in Chinese population: a meta-analysis based on 26,810 subjects

SUPPLEMENTARY MATERIALS

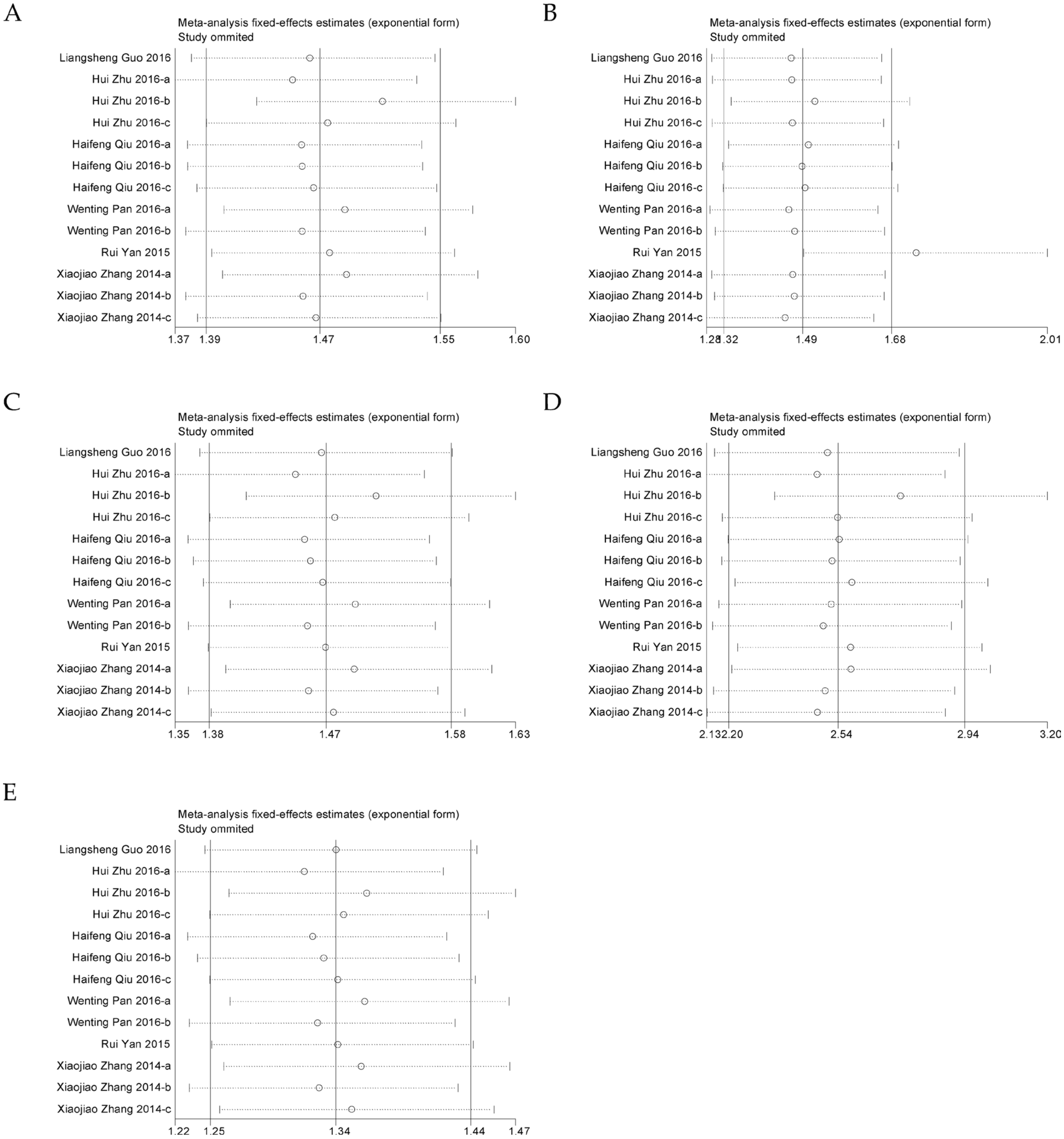

Supplementary Figure 1: Sensitivity analyses for *HOTAIR* rs920778 polymorphism and cancer. (**A**) allele model (T vs. C); (**B**) recessive model (TT vs. TC + CC); (**C**)dominant model (TC + TT vs. CC); (**D**) homozygous model (TT vs. CC); (**E**) heterozygous model (TC vs. CC).

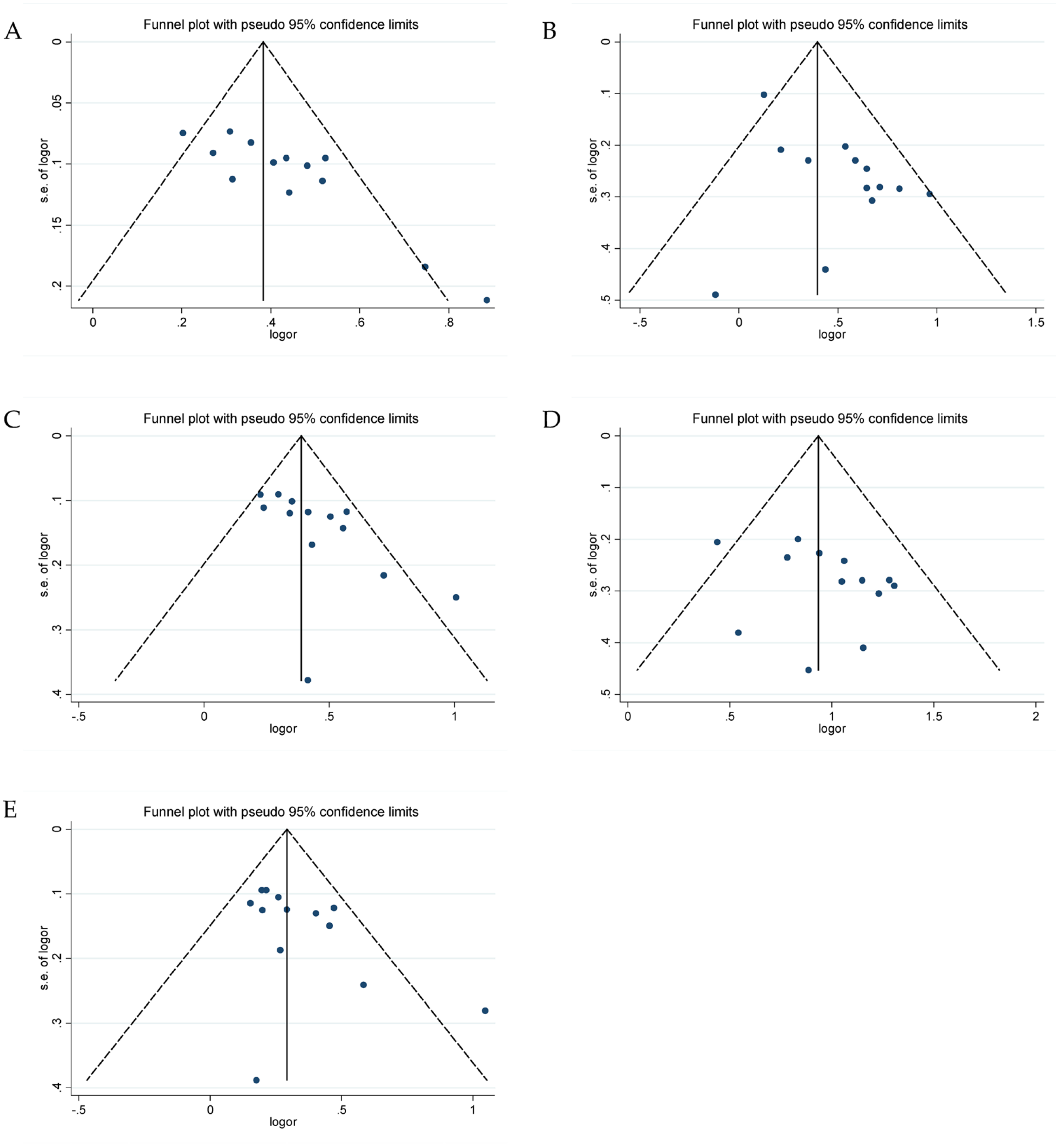

Supplementary Figure 2: Funnel plots for *HOTAIR* rs920778 polymorphism and cancer. (**A**) allele model (T vs. C); (**B**) recessive model (TT vs. TC + CC); (**C**) dominant model (TC + TT vs. CC); (**D**) homozygous model (TT vs. CC); (**E**) heterozygous model (TC vs. CC).

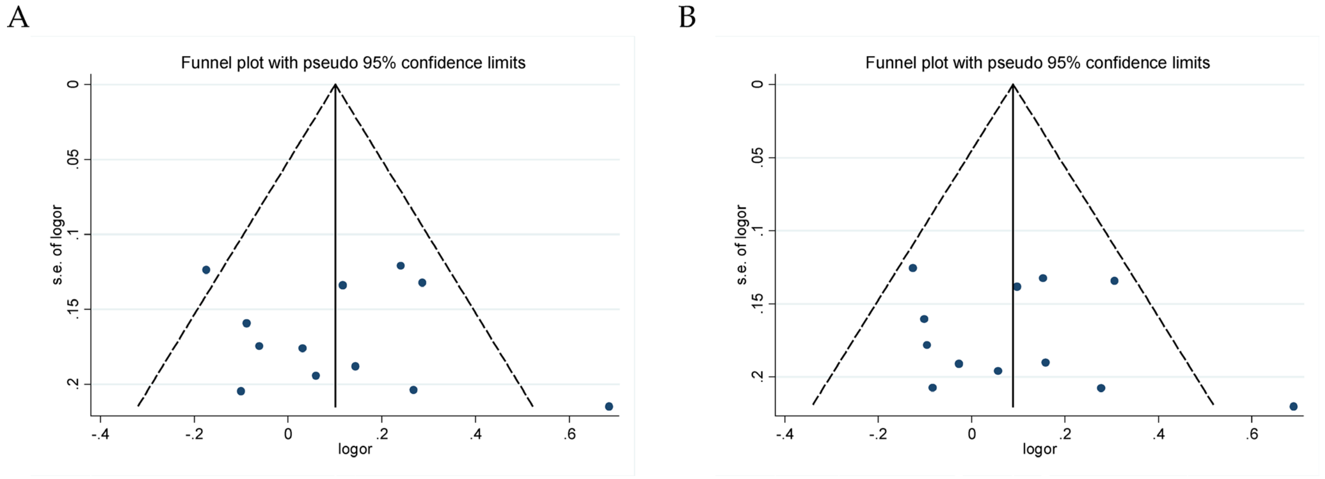

Supplementary Figure 3: Funnel plots for *HOTAIR* rs4759314 polymorphism and cancer. (**A**) dominant model (GA + GG vs. AA); (**B**) heterozygous model (GA vs. AA).

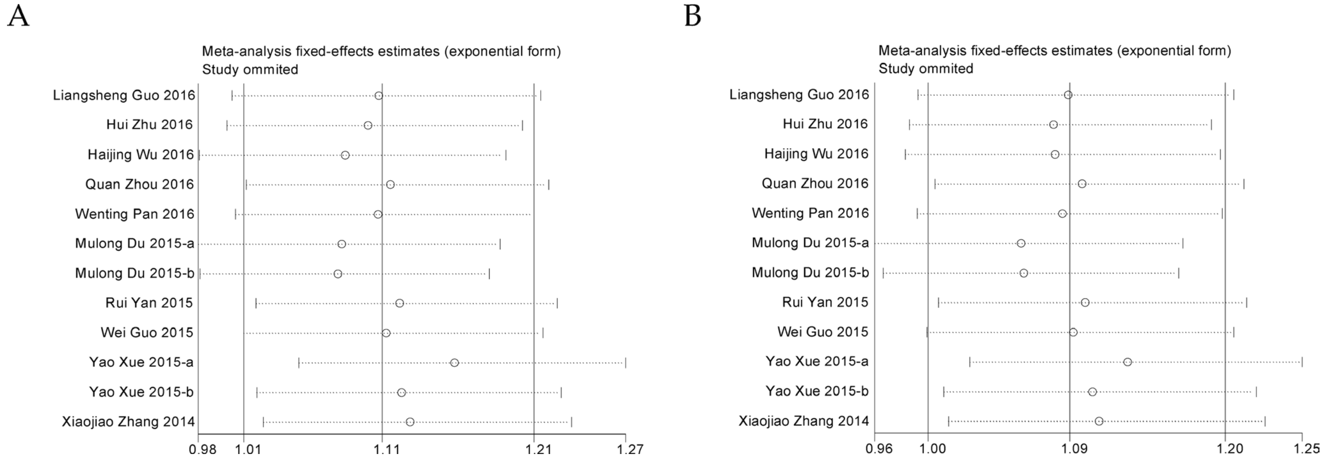

Supplementary Figure 4: Sensitivity analyses for *HOTAIR* rs4759314 polymorphism and cancer. (**A**) dominant model (GA + GG vs. AA); (**B**) heterozygous model (GA vs. AA).

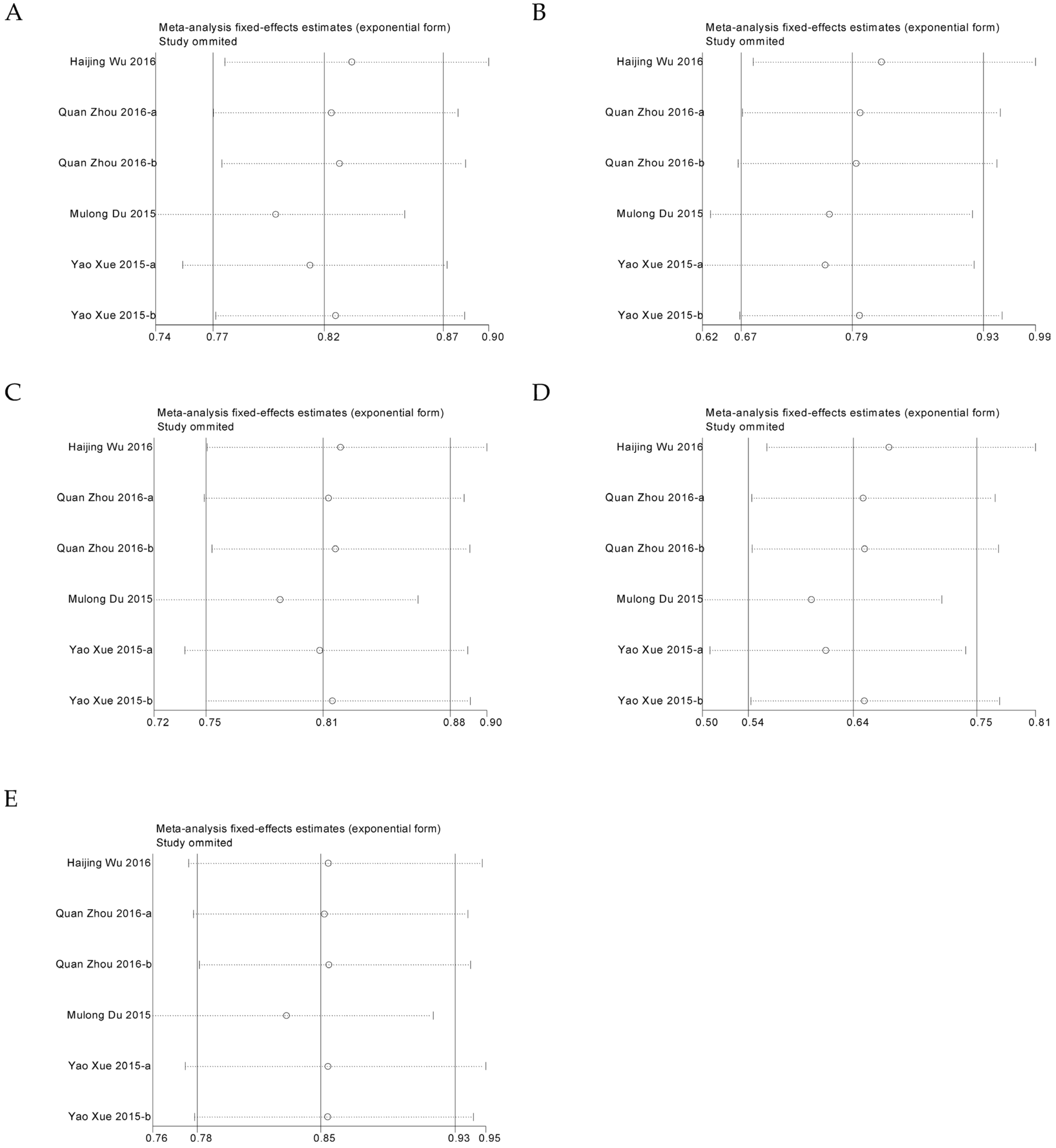

Supplementary Figure 5: Sensitivity analyses for *HOTAIR* rs7958904 polymorphism and cancer. (**A**) allele model (C vs. G); (**B**) recessive model (CC vs. CG + GG); (**C**) dominant model (CG + CC vs. GG); (**D**) homozygous model (CC vs. GG); (**E**) heterozygous model (CG vs. GG).

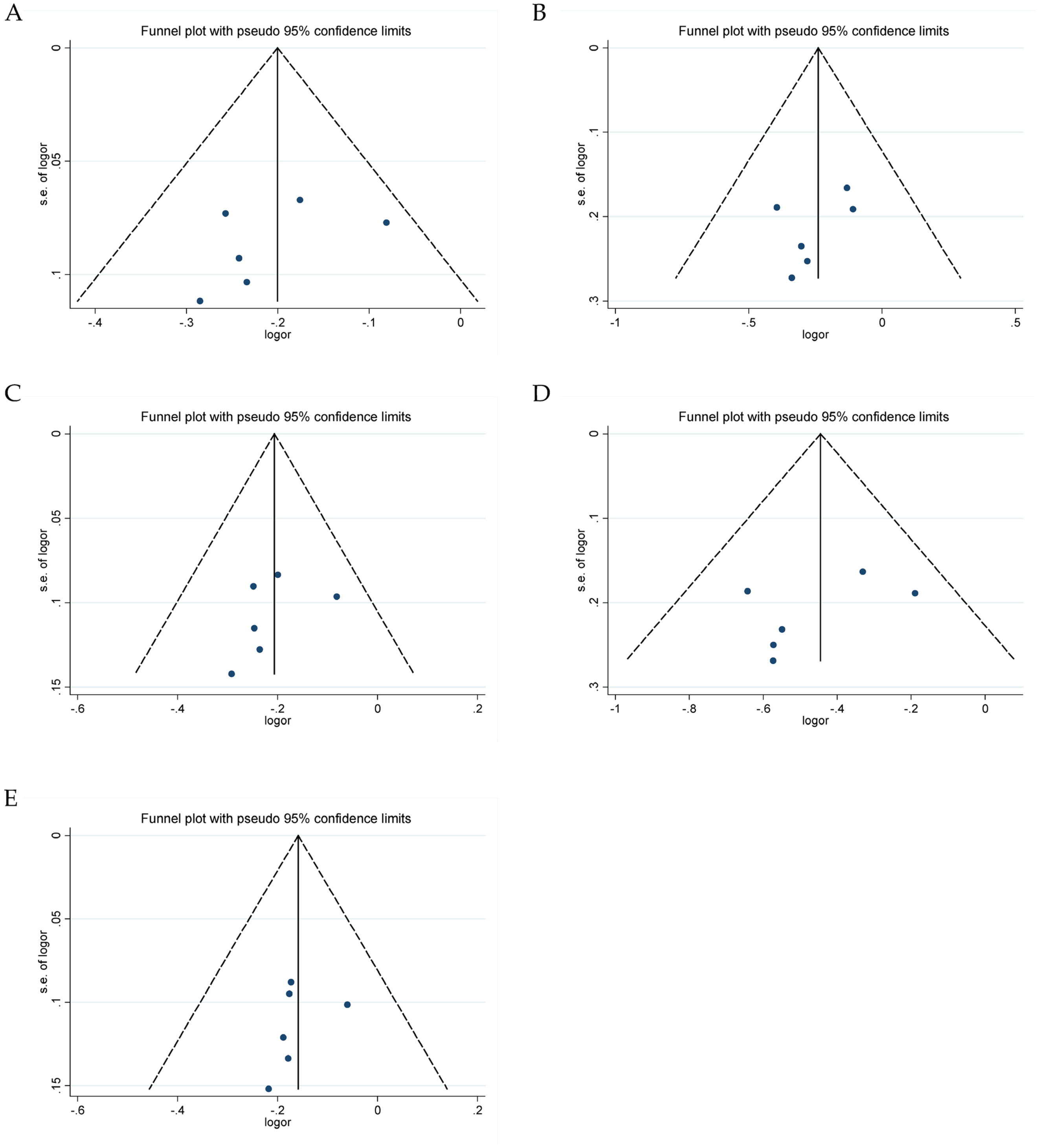

Supplementary Figure 6: Funnel plots for *HOTAIR* rs7958904 polymorphism and cancer. (**A**) allele model (C vs. G); (**B**) recessive model (CC vs. CG + GG); (**C**) dominant model (CG + CC vs. GG); (**D**) homozygous model (CC vs. GG); (**E**) heterozygous model (CG vs. GG).

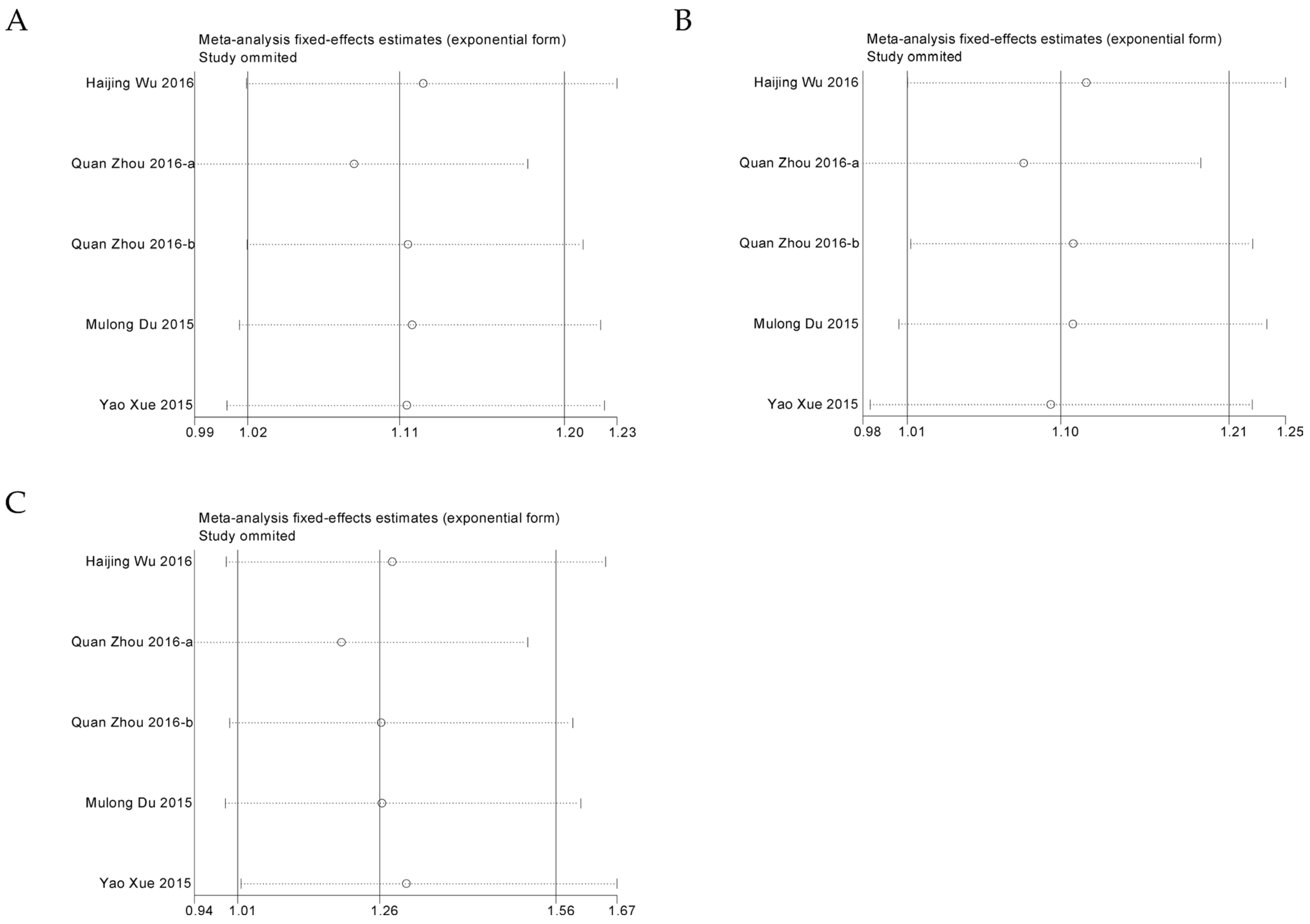

Supplementary Figure 7: Sensitivity analyses for *HOTAIR* rs874945 polymorphism and cancer. (**A**) allele model (A vs. G); (**B**) dominant model (AG + AA vs. GG); (**C**) homozygous model (AA vs. GG).

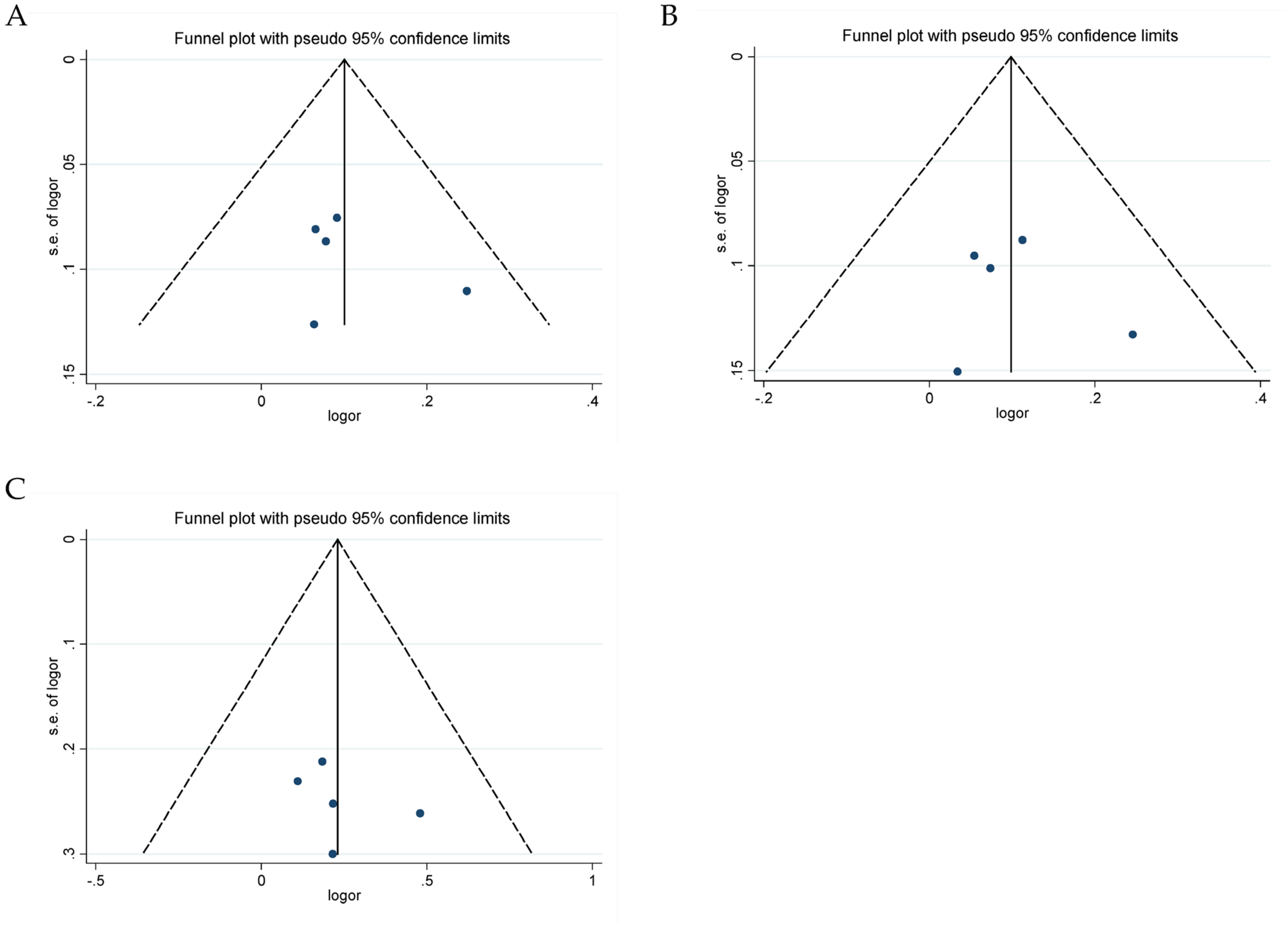

Supplementary Figure 8: Funnel plots for *HOTAIR* rs874945 polymorphism and cancer. (**A**) allele model (A vs. G); (**B**) dominant model (AG + AA vs. GG); (**C**) homozygous model (AA vs. GG).

Supplementary Table 1: Characteristics of case-control studies on *HOTAIR* polymorphisms and cancer risk included in the meta-analysis. See Supplementary_Table_1
