# Supplementary material for: Quantitative assessment of lncRNA HOTAIR polymorphisms and cancer risk in Chinese population: a meta-analysis based on 26,810 subjects
Source: Oncotarget. 2017 Aug 1;8(35):59698–708. doi: 10.18632/oncotarget.19776 (PMC5601769; doi:10.18632/oncotarget.19776)
Supplement: Supplementary file 2 [file oncotarget-08-59698-s002.docx]

**Supplementary Table 1: Characteristics of case-control studies on *HOTAIR* polymorphisms and cancer risk included in the meta-analysis**

| **First author** | **Year** | **Source of controls** | **Case** | **Control** | **Genotype distribution** | | | | | | **Genotyping methods** | **Age and sex matched** | **Type of cancers** | ***P* for HWE^a^** |
| --- | --- | --- | --- | --- | --- | --- | --- | --- | --- | --- | --- | --- | --- | --- |
|  |  |  |  |  | **Case** | | | **Control** | | |  |  |  |  |
| rs920778 C＞T | | | | | TT | TC | CC | TT | TC | CC |  | | | |
| Guo LS | 2016 | Hospital | 510 | 713 | 52 | 189 | 269 | 30 | 235 | 448 | MALDI-TOF-MS | matched | cervical cancer | 0.907 |
| Zhu H-a | 2016 | NA | 600 | 600 | 53 | 259 | 288 | 19 | 209 | 372 | RFLP | matched | thyroid carcinoma | 0.109 |
| Zhu H-b | 2016 | NA | 1000 | 1000 | 62 | 385 | 553 | 44 | 348 | 608 | RFLP | matched | thyroid carcinoma | 0.513 |
| Zhu H-c | 2016 | NA | 800 | 800 | 68 | 316 | 416 | 31 | 284 | 485 | RFLP | matched | thyroid carcinoma | 0.181 |
| Qiu HF-a | 2016 | Hospital | 139 | 300 | 10 | 32 | 97 | 11 | 30 | 259 | Taqman | matched | ovarian cancer | 0.000 |
| Qiu HF-b | 2016 | Hospital | 190 | 380 | 15 | 37 | 138 | 11 | 48 | 321 | Taqman | matched | ovarian cancer | 0.000 |
| Qiu HF-c | 2016 | NA | 215 | 430 | 47 | 78 | 90 | 54 | 150 | 226 | Taqman | matched | cervical cancer | 0.000 |
| Pan WT-a | 2016 | Population | 500 | 1000 | 31 | 194 | 275 | 24 | 368 | 608 | RFLP | matched | gastric cancer | 0.000 |
| Pan WT-b | 2016 | Population | 300 | 600 | 28 | 127 | 145 | 21 | 207 | 372 | RFLP | matched | gastric cancer | 0.230 |
| Yan R | 2015 | Population | 502 | 504 | 339 | 151 | 12 | 296 | 190 | 18 | RFLP | matched | breast cancer | 0.748 |
| Zhang XJ-a | 2014 | Population | 1000 | 1000 | 83 | 389 | 528 | 41 | 358 | 601 | RFLP | matched | esophageal cancer | 0.173 |
| Zhang XJ-b | 2014 | Hospital | 510 | 550 | 47 | 207 | 256 | 20 | 186 | 344 | RFLP | matched | esophageal cancer | 0.401 |
| Zhang XJ-c | 2014 | Population | 588 | 600 | 51 | 203 | 307 | 17 | 205 | 378 | RFLP | matched | esophageal cancer | 0.082 |
| rs4759314 A＞G | | | | | GG | GA | AA | GG | GA | AA |  | | | |
| Guo LS | 2016 | Hospital | 510 | 713 | 11 | 121 | 378 | 11 | 158 | 544 | MALDI-TOF-MS | matched | cervical cancer | 0.903 |
| Zhu H-a | 2016 | NA | 600 | 600 | 2 | 58 | 540 | 2 | 45 | 553 | RFLP | matched | thyroid carcinoma | 0.297 |
| Wu HJ | 2016 | NA | 1000 | 1000 | 41 | 140 | 819 | 23 | 125 | 852 | MALDI-TOF-MS | matched | ovarian cancer | 0.000 |
| Zhou Q-a | 2016 | Hospital | 500 | 500 | 15 | 62 | 423 | 11 | 64 | 425 | MALDI-TOF-MS | matched | osteosarcoma | 0.000 |
| Pan WT | 2016 | Population | 500 | 1000 | 1 | 48 | 451 | 3 | 83 | 914 | RFLP | matched | gastric cancer | 0.448 |
| Du ML-a | 2015 | Hospital | 753 | 1057 | 3 | 126 | 624 | 6 | 136 | 915 | Taqman | matched | gastric cancer | 0.699 |
| Du ML-b | 2015 | Hospital | 522 | 589 | 3 | 60 | 459 | 2 | 36 | 549 | Taqman | matched | gastric cancer | 0.098 |
| Yan R | 2015 | Population | 502 | 504 | 1 | 50 | 451 | 2 | 54 | 448 | RFLP | matched | breast cancer | 0.785 |
| Guo W | 2015 | Population | 515 | 654 | 1 | 53 | 461 | 1 | 64 | 589 | RFLP | unmatched | gastric cancer | 0.587 |
| Xue Y-a | 2015 | Hospital | 1147 | 1203 | 1 | 135 | 1011 | 9 | 157 | 1037 | Taqman | matched | colorectal cancer | 0.260 |
| Xue Y-b | 2015 | Hospital | 587 | 652 | 4 | 65 | 517 | 2 | 79 | 571 | Taqman | matched | colorectal cancer | 0.673 |
| Zhang XJ-a | 2014 | Population | 1000 | 1000 | 2 | 81 | 917 | 1 | 89 | 910 | RFLP | matched | esophageal cancer | 0.436 |
| rs7958904 G＞C | | | | | CC | CG | GG | CC | CG | GG |  | | | |
| Wu HJ | 2016 | NA | 1000 | 1000 | 51 | 355 | 594 | 87 | 380 | 533 | MALDI-TOF-MS | matched | ovarian cancer | 0.105 |
| Zhou Q-a | 2016 | Hospital | 500 | 500 | 25 | 180 | 295 | 40 | 194 | 266 | MALDI-TOF-MS | matched | osteosarcoma | 0.580 |
| Zhou Q-b | 2016 | Hospital | 400 | 400 | 31 | 140 | 229 | 48 | 152 | 200 | MALDI-TOF-MS | matched | osteosarcoma | 0.025 |
| Du ML | 2015 | Hospital | 753 | 1057 | 51 | 276 | 412 | 85 | 404 | 568 | Taqman | matched | gastric cancer | 0.271 |
| Xue Y-a | 2015 | Hospital | 1147 | 1203 | 74 | 399 | 672 | 99 | 456 | 646 | Taqman | matched | colorectal | 0.147 |
| Xue Y-b | 2015 | Hospital | 587 | 652 | 33 | 206 | 347 | 57 | 248 | 346 | Taqman | matched | colorectal | 0.192 |
| rs874945 G＞A | | | | | AA | AG | GG | AA | AG | GG |  | | | |
| Wu HJ | 2016 | NA | 1000 | 1000 | 52 | 283 | 665 | 44 | 279 | 677 | MALDI-TOF-MS | matched | ovarian cancer | 0.029 |
| Zhou Q-a | 2016 | Hospital | 500 | 500 | 40 | 150 | 310 | 27 | 135 | 338 | MALDI-TOF-MS | matched | osteosarcoma | 0.008 |
| Zhou Q-b | 2016 | Hospital | 400 | 400 | 27 | 106 | 267 | 22 | 108 | 270 | MALDI-TOF-MS | matched | osteosarcoma | 0.014 |
| Du ML | 2015 | Hospital | 753 | 1057 | 31 | 225 | 495 | 36 | 307 | 714 | Taqman | matched | gastric cancer | 0.672 |
| Xue Y-a | 2015 | Hospital | 1147 | 1203 | 40 | 356 | 751 | 39 | 346 | 817 | Taqman | matched | colorectal | 0.749 |
| rs1899663 G＞T | | | | | TT | TG | GG | TT | TG | GG |  | | | |
| Guo LS | 2016 | Hospital | 510 | 713 | 8 | 146 | 356 | 13 | 191 | 509 | MALDI-TOF-MS | matched | cervical cancer | 0.308 |
| Zhu H-a | 2016 | NA | 600 | 600 | 7 | 151 | 442 | 12 | 175 | 413 | RFLP | matched | thyroid carcinoma | 0.184 |
| Pan WT | 2016 | Population | 500 | 1000 | 6 | 118 | 376 | 13 | 255 | 732 | RFLP | matched | gastric cancer | 0.078 |
| Yan R | 2015 | Population | 502 | 504 | 14 | 149 | 339 | 20 | 158 | 326 | RFLP | matched | breast cancer | 0.876 |
| Zhang XJ-a | 2014 | Population | 1000 | 1000 | 19 | 256 | 725 | 26 | 250 | 724 | RFLP | matched | esophageal cancer | 0.430 |

**HWE: Hardy-Weinberg Equilibrium. ^a^HWE in control. NA: not available. MALDI-TOF-MS: Matrix-Assisted Laser Desorption/ Ionization Time of Flight Mass Spectrometry.**

**RFLP: Restriction Fragment Length Polymorphism.**
